# Is the Sex Instinct the Strongest?

**Published:** 1914-09

**Authors:** 


### Is the Sex Instinct the Strongest?

It is generally regarded by most people that sex is the strongest of all our instincts, and with not a few the idea prevails that "life is one long sex pursuit," meaning thereby that sex dominates and rules our every motive and action. Whether this is true depends on the interpretation placed upon what constitutes self-control. Is it intelligence; i. e., discrimination and judgment in determining what is good or bad for the individual, or is it a blind groping instinct purposely endowed with persistency to assure propagating? In other words, does the instinct to propagate act independently of intelligence? We see that this is true in brute creation. But man is possessed with the mental faculty for the purpose of making himself a responsible being. Therefore, in the very nature of things, intelligence *must* control all functions of the body, just as a general has command over the different divisions of his army. But how far a man *is* controlled by his intelligence is another question. If his mental balance is presumably weak, and an instinctive force is proportionately strong, there is little doubt but that the instinct will influence the action of the mental function. And when through inherent weakness and the influence of environment the mental element becomes diseased and obsessed with sex ideas there is no doubt as to the dominant power. The fact that a large class of society is being dominated by sex is not an argument that sex is the stronger influence, but rather an expression of a condition without explanation of why it is so.

Again, judgment itself is founded on the experience and custom of society. For example, man's moral conduct based on his judgment will be influenced by the attitude he takes regarding the question whether or not sexual relation is essential to health. Therefore, conduct under normal conditions and influence is a matter of moral control, and where it is otherwise it is an indication of disease and weakness in inhibiting the normal functions of the intellect. The fact is there is much disease and weakness in a man's life, and the instinct of propagation being strong, it is an easy matter to permit it to subjugate other influences, and in this manner it may appear to be the most dominant.

Here, again, we must call in the aid of eugenics to build up an inherent strain of characters that will compel moral action, because it is most natural to be so.

